# The Novel P_II_-Interacting Protein PirA Controls Flux into the Cyanobacterial Ornithine-Ammonia Cycle

**DOI:** 10.1128/mBio.00229-21

**Published:** 2021-03-23

**Authors:** Paul Bolay, Rokhsareh Rozbeh, M. Isabel Muro-Pastor, Stefan Timm, Martin Hagemann, Francisco J. Florencio, Karl Forchhammer, Stephan Klähn

**Affiliations:** a Helmholtz Centre for Environmental Research, Department of Solar Materials, Leipzig, Germany; b Interfaculty Institute for Microbiology and Infection Medicine, Organismic Interactions Department, Tübingen University, Tübingen, Germany; c Instituto de Bioquímica Vegetal y Fotosíntesis, CSIC-Universidad de Sevilla, Sevilla, Spain; d Department of Plant Physiology, University of Rostock, Rostock, Germany; John Innes Centre; Yale School of Medicine

**Keywords:** nitrogen metabolism, cyanobacteria, small inhibitory proteins, P_II_ protein

## Abstract

Among prokaryotes, cyanobacteria have an exclusive position as they perform oxygenic photosynthesis. Cyanobacteria substantially differ from other bacteria in further aspects, e.g., they evolved a plethora of unique regulatory mechanisms to control primary metabolism. This is exemplified by the regulation of glutamine synthetase (GS) via small proteins termed inactivating factors (IFs). Here, we reveal another small protein, encoded by the *ssr0692* gene in the model strain *Synechocystis* sp. PCC 6803, that regulates flux into the ornithine-ammonia cycle (OAC), the key hub of cyanobacterial nitrogen stockpiling and remobilization. This regulation is achieved by the interaction with the central carbon/nitrogen control protein P_II_, which commonly controls entry into the OAC by activating the key enzyme of arginine synthesis, *N*-acetyl-l-glutamate kinase (NAGK). In particular, the Ssr0692 protein competes with NAGK for P_II_ binding and thereby prevents NAGK activation, which in turn lowers arginine synthesis. Accordingly, we termed it P_II_-interacting regulator of arginine synthesis (PirA). Similar to the GS IFs, PirA accumulates in response to ammonium upshift due to relief from repression by the global nitrogen control transcription factor NtcA. Consistent with this, the deletion of *pirA* affects the balance of metabolite pools of the OAC in response to ammonium shocks. Moreover, the PirA-P_II_ interaction requires ADP and is prevented by P_II_ mutations affecting the T-loop conformation, the major protein interaction surface of this signal processing protein. Thus, we propose that PirA is an integrator determining flux into N storage compounds not only depending on the N availability but also the energy state of the cell.

## Abstract (precis)

Cyanobacteria contribute a significant portion to the annual oxygen yield and play important roles in biogeochemical cycles, e.g., as major primary producers. Due to their photosynthetic lifestyle, cyanobacteria also arouse interest as hosts for the sustainable production of fuel components and high-value chemicals.

## INTRODUCTION

Nitrogen (N) is one of the key elements of life and needs to be incorporated into biomolecules via assimilatory pathways. Despite being an ever-present resource in the atmosphere, only a few bacteria can fix dinitrogen (N_2_), and the majority rely on the uptake and assimilation of combined N sources from their environment (1–3). To respond to fluctuations in the availability of combined N sources, bacteria possess complex regulatory networks to control N uptake as well as the activity of assimilatory enzymes (for reviews, see references 4–7). As a prime example, glutamine synthetase (GS), a key enzyme of bacterial ammonium assimilation, is tightly regulated in a variety of ways. In Escherichia coli and other proteobacteria, the expression of the GS-encoding *glnA* gene is controlled at the transcriptional level by the widespread NtrC/NtrB two-component system (7). Moreover, GS is controlled at the activity level via cumulative feedback inhibition from numerous metabolites related to N and energy metabolism as well as by covalent modification, i.e., adenylylation of the GS subunits. This modification system is operated by a bicyclic modification cascade involving the ubiquitous P_II_ signal transducer protein as a regulatory element (reviewed in reference 8). However, striking differences compared to widely accepted paradigms of N assimilation have been revealed in other bacteria, e.g., cyanobacteria.

Cyanobacteria are the only prokaryotes performing oxygenic photosynthesis and play a major role in global biogeochemical cycles (9–14). Presently they are receiving growing interest as biocatalysts in photobiotechnological applications, e.g., for the sustainable production of valued chemicals and fuels (15–19). To rationally engineer cyanobacteria, i.e., channeling metabolic fluxes to obtain the maximum yield of a desired chemical product, it is of paramount importance to fully comprehend underlying regulatory processes targeting primary metabolism. Although our overall understanding of cyanobacterial systems is still fragmentary compared to other well-established bacterial models, a few systems have been extensively investigated and include distinctive features. For instance, GS activity is controlled via the interaction with small, inhibitory proteins unique to cyanobacteria (20, 21). These GS-inactivating factors (IFs) exclusively control GS activity linearly with their abundance. Moreover, with the global nitrogen control protein NtcA, cyanobacteria use another type of transcription factor to control the expression of genes in response to N fluctuation (22). NtcA belongs to the CRP transcriptional regulator family and commonly works as an activator of N assimilatory genes (23–26). During N limitation, increasing levels of 2-oxoglutarate and the coactivator protein PipX stimulate DNA binding of NtcA (27–29). The interaction between NtcA and PipX is antagonized by the P_II_ protein, which acts as a global multitasking sensor and regulator, adjusting the carbon-nitrogen homeostasis through versatile protein-protein interactions (30, 31). This, for instance, includes the key enzyme for arginine synthesis, N-acetyl glutamate kinase (NAGK), which is activated by complex formation with P_II_ (32).

In addition to the activation of N assimilatory genes, NtcA can also act as a repressor of genes under N limitation. The physiological consequences of simultaneous positive and negative transcriptional regulation are again exemplified by the well-investigated GS regulatory system. Under N-limiting conditions, NtcA activates the transcription of the *glnA* gene, thereby increasing GS abundance and the rate of ammonium assimilation. Simultaneously, enhanced DNA binding of NtcA represses the transcription of the genes *gifA* and *gifB*, encoding the two known IFs, IF7 and IF17 (33). GS activity thereby is tuned in a trade-off between cellular N demands and relief from the metabolic burden imposed by the glutamate- and ATP-consuming GS-catalyzed reaction (for a review, see reference 88). Besides *gifA* and *gifB*, only a few other genes appear to be negatively regulated by NtcA. In an attempt to define the entire regulon of NtcA in the unicellular model strain *Synechocystis* sp. strain PCC 6803 (here called *Synechocystis*), Giner-Lamia et al. identified the gene *ssr0692* as another NtcA-repressed candidate (23). It encodes a small protein consisting of 51 amino acids with a high portion of N-rich, positively charged residues that were shown to be indispensable for protein-protein interaction in the case of the GS IFs (21). These distinguishing traits point toward a vital function related to N control similar to the known GS IFs, e.g., as a regulator of a metabolic pathway.

Here, we report on the functional analysis of the small protein Ssr0692 in *Synechocystis*. It accumulates in response to ammonium supply and fulfills crucial regulatory roles in cyanobacterial metabolism via interaction with the P_II_ signaling protein. We show that it directly interferes with the P_II_-dependent activation of NAGK. Consistent with this, under fluctuating N regimes, *ssr0692* mutant strains are impaired in balancing the synthesis of arginine and other amino acids associated with the cyanobacterial ornithine ammonia cycle identified recently (34). Therefore, we named Ssr0692 the P_II_-interacting regulator of arginine synthesis (PirA).

## RESULTS

**FIG 1:**
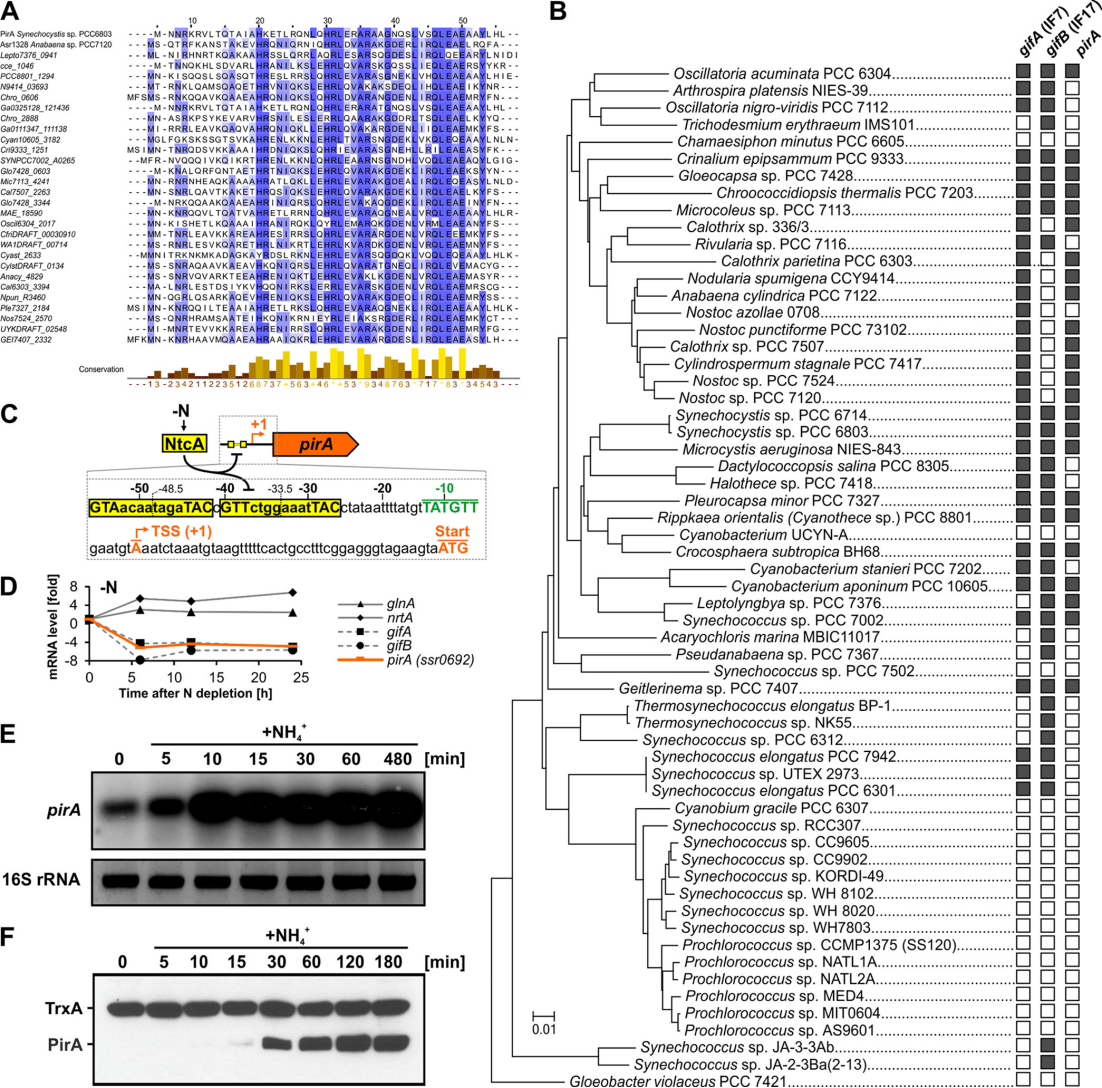
N-regulated gene *pirA* and its occurrence among cyanobacteria. (A) Amino acid alignment of randomly selected cyanobacterial PirA homologs. The alignment was made using ClustalW and visualized by using Jalview. (B) Phylogenetic tree of selected cyanobacteria based on 16S rRNA gene sequences. The tree was generated with the MEGA7 (83) software package and the neighbor-joining method. Note that we reused a calculated tree from our previous publication (38) and assigned the presence of genes in the corresponding genomes manually. Gene presence (illustrated by filled rectangles) was investigated using the BLASTP algorithm (84). As a reference, the amino acid sequences of PirA, IF7 (GifA, Ssl1911), and IF17 (GifB, Sll1515) from *Synechocystis* were used. (C) Overview of the promoter region upstream of the *pirA* gene in *Synechocystis*. Two putative NtcA binding sites are highlighted. The transcriptional start site (TSS; +1) and the location of the −10 element were extracted from differential transcriptome sequencing data (85). (D) Changes of mRNA levels for several *Synechocystis* genes in response to N limitation. Data were extracted and plotted from previously published microarray data (86). (E) Northern blot showing transcript accumulation of *pirA* in nitrate-grown *Synechocystis* cells upon addition of 10 mM ammonium chloride. 16S rRNA was used as a loading control. (F) Western blot showing changes in PirA protein levels in response to ammonium upshifts. For this, a specific, customized antibody against PirA was raised in rabbit. An antibody against thioredoxin (TrxA) was used to verify equal loading.

Homologs of the *pirA* gene of *Synechocystis* are frequently present in cyanobacterial genomes and show a high degree of sequence conservation at the amino acid level (Fig. 1A and B). With only a few exceptions, sequences similar to that of PirA are absent from genomes of other bacterial phyla (as of July 2020, exceptions are “*Candidatus* Gracilibacteria bacterium,” *Chloroflexaceae bacterium*, *Flavobacterium* sp. strain CLA17, and *Methylacidiphilales bacterium*). At first glance, this observation suggests a function associated with oxygenic photosynthesis. However, *pirA* has previously been identified as part of the NtcA regulon in *Synechocystis* (23), consistent with two putative NtcA binding motifs located upstream of the transcriptional start site (TSS) (Fig. 1C). In promoters that are activated by NtcA, the respective binding motifs are centered close to position −41.5 with regard to the TSS (+1), bringing NtcA into a favorable position to promote the binding of RNA polymerase (35). However, the location of both motifs present in the *pirA* promoter is compatible with a repressive role of NtcA in the transcription of this gene. The proximal site, which is centered at −33.5 bp upstream of the TSS, is in a position very similar to the binding sites described for the well-characterized NtcA-repressed *gifA-B* genes (23, 33). NtcA binding in close proximity to the TSS would mediate repression by steric hindrance of the RNA polymerase interaction. This assumption is consistent with *pirA* downregulation under N limitation, similar to the *gifA-B* genes and in contrast to NtcA-activated genes such as *glnA* or *nrtA*, encoding GS and a nitrate transporter component, respectively (Fig. 1D). Interestingly, the distal site, centered at −48.5 bp, could also interfere with the polymerase binding, specifically by preventing the correct interaction of the carboxy-terminal domain of its alpha subunit with the promoter (36). The presence of two NtcA binding motifs probably contributes to a tighter control of *pirA* expression as a function of N conditions.

### PirA accumulates under N excess and is linked to a function in cyanobacterial N metabolism.

Genes that are repressed by NtcA, such as the *gifA-B* genes, show low or even nondetectable transcription under N limitation but are highly expressed in response to excess N supply. To test whether this is also true for *pirA*, we precultivated *Synechocystis* cells in the presence of nitrate and analyzed transcript levels after induction of N excess by adding 10 mM ammonium. As expected, the *pirA* mRNA strongly accumulated under these conditions (Fig. 1E). To investigate whether this regulatory pattern is conveyed to the protein level, we obtained an antibody specific to the PirA protein. Consistent with the observed transcriptional regulation, the PirA protein also accumulated in response to ammonium upshifts (Fig. 1F). Moreover, the protein appeared to have a high turnover because it eluded detection shortly after N was depleted (see Fig. S1 in the supplemental material). These observations clearly link PirA and its function to cyanobacterial N metabolism.

10.1128/mBio.00229-21.3 FIG S1 Western blot showing changes in PirA protein levels in response to ammonium upshifts and subsequent N depletion. For this, a specific, customized antibody against PirA has been raised in rabbit. An antibody against thioredoxin (TrxA) was used to verify equal loading. Download FIG S1, TIF file, 0.1 MB. Copyright © 2021 Bolay et al. 2021 Bolay et al. https://creativecommons.org/licenses/by/4.0/ This content is distributed under the terms of the Creative Commons Attribution 4.0 International license.

**FIG 2:**
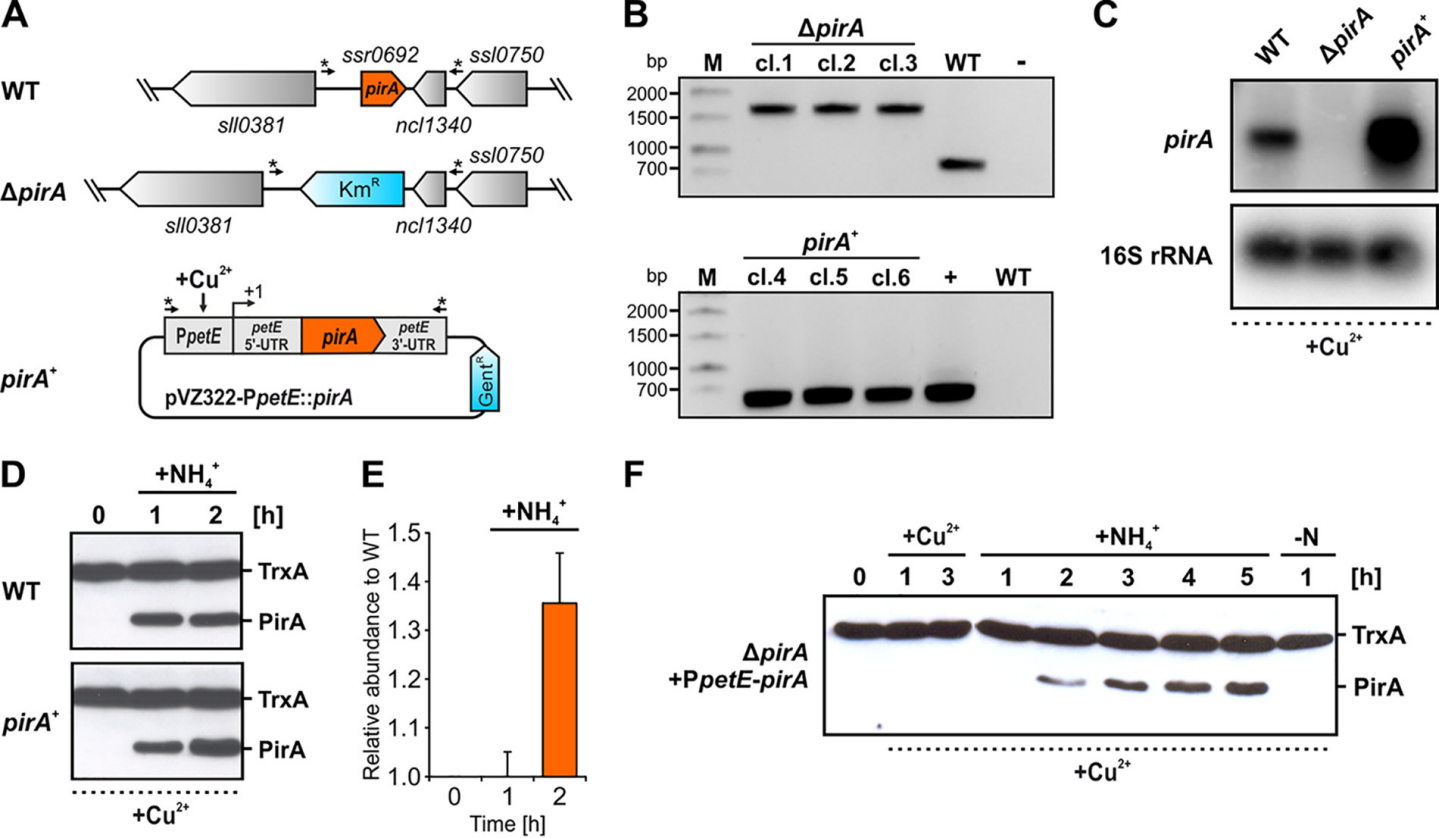
Properties and expression profiles in Δ*pirA* and *pirA*^+^ recombinant strains. (A) Schematic view of the *pirA* locus in the WT and in the Δ*pirA* knockout strain as well as of a pVZ322 plasmid derivative harboring a *pirA* gene copy under the control of the Cu^2+^-inducible promoter P*petE* that is present in the *pirA*^+^ overexpression strain. In the Δ*pirA* knockout strain, *pirA* was replaced by a kanamycin resistance cassette (Km^r^) via homologous recombination. The plasmid enabling ectopic *pirA* expression was introduced into *Synechocystis* WT. The arrows labeled with asterisks indicate the binding sites for primers used to verify the mutants. (B) PCR verification of the genotype of independently obtained mutant strains. In each case three clones were tested using primer combinations Ssr0692_KO-seg_fw/Ssr0692_KO-seg_rev (in case of Δ*pirA* strain) or P*petE*_fw(XhoI) and Toop_rev(AseI) (in case of *pirA*^+^ strain). M, marker; bp, base pairs; cl., clone; −, negative control (water as the template); +, positive control (purified plasmid as the template). (C) Relative abundance of the *pirA* mRNA, measured via Northern blotting using sequence-specific ^32^P-labeled ssRNA probes. In all cases, RNA was isolated from cells grown in the presence of 1 μM CuSO_4_. (D) Western blot showing PirA protein levels in cells of the WT and *pirA*^+^ strains, treated with 1 μM Cu^2+^ for 3 h and afterwards with 10 mM ammonium. Thioredoxin (TrxA) levels verify equal loading. (E) PirA levels relative to WT. Data were obtained by densitometric evaluation of respective bands using the ImageJ software (87). Data are mean ± standard deviation (SD) values obtained from two independent Western blots, i.e., two biological replicates (independent clones). (F) PirA accumulation in a Δ*pirA* strain that was complemented with a *pirA* gene fused to the *petE* promoter. Note that the data shown here were obtained using a mutant in which the P*petE-pirA* construct was integrated into the chromosome, i.e., this strain does not harbor the plasmid derivative shown in panel A.

To investigate the biological function of PirA, knockout and overexpression strains for the *pirA* gene were established in *Synechocystis*. The Δ*pirA* knockout mutant was generated by replacing the entire *pirA* open reading frame with a kanamycin resistance cassette via homologous recombination. In the case of the *pirA*^+^ overexpression strain, a pVZ322 plasmid derivative harboring a transcriptional fusion of *pirA* with the Cu^2+^-inducible *petE* promoter (P*petE*) was transferred into the *Synechocystis* wild type (WT) (Fig. 2A). Full segregation of the mutant allele in the Δ*pirA* mutant as well as the presence of the recombinant plasmid in the *pirA*^+^ strain were verified by PCR (Fig. 2B). Subsequent Northern blot analyses with RNA isolated from cells grown in the presence of 1 μM CuSO_4_ confirmed the generated mutants: the overexpression strain showed increased *pirA* mRNA levels compared to the WT, while in the knockout strain the *pirA* transcript was absent (Fig. 2C). Interestingly, even though the mRNA was present and its abundance significantly increased in the *pirA*^+^ strain due to the ectopic expression triggered by Cu^2+^, the PirA protein could not be detected in nitrate-grown cells. However, after adding ammonium, which triggers the expression of the native *pirA* gene from the chromosome, increased PirA levels were detectable in the *pirA*^+^ strain compared to the WT (Fig. 2D). This, in addition to the verified increase at the mRNA level, clearly confirmed that the overexpression construct is operative. Obviously, PirA abundance is not exclusively controlled at the transcriptional level. This observation was further supported by experiments using a *pirA* knockout mutant in which a P*petE*-fused gene copy was introduced. As observed before, the PirA protein could not be detected after adding Cu^2+^ to nitrate-grown cells (Fig. 2E). Remarkably, its presence was still N dependent, similar to the WT, i.e., it was detectable only after adding ammonium (Fig. 2E), even though *pirA* transcription was controlled by P*petE* and, hence, exclusively triggered by Cu^2+^. Consequently, these data indicate an additional, posttranscriptional control mechanism, which obviously prevents stable PirA accumulation unless N availability suddenly increases. This again resembles the GS IFs encoded by the *gifA-B* genes, which are tightly regulated at the transcriptional as well as posttranscriptional level (37–39).

### PirA plays a critical role upon changes in the C/N balance.

**FIG 3:**
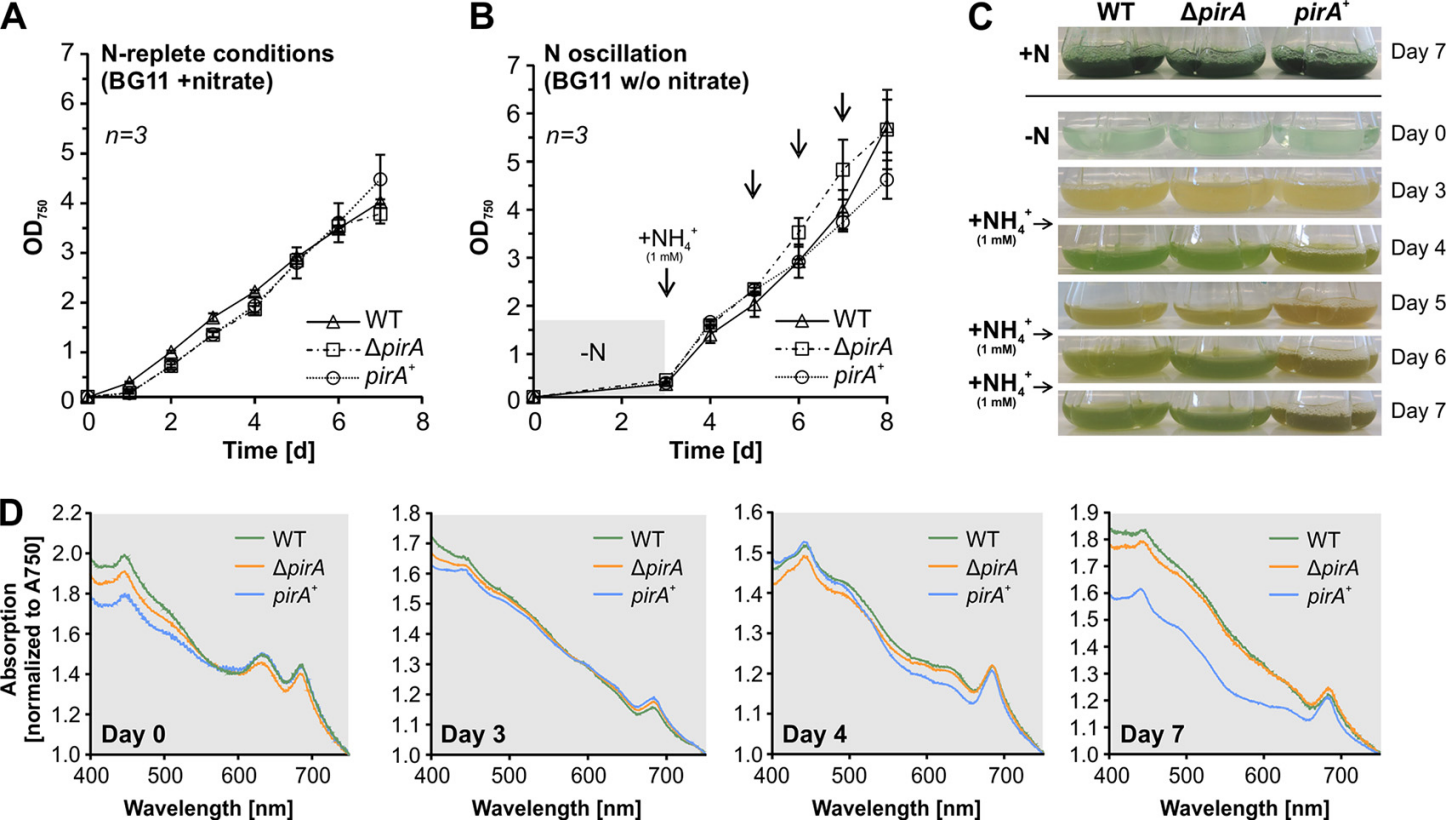
Growth and pigmentation of the WT and the Δ*pirA* and *pirA*^+^ mutant strains when N is oscillating. (A and B) Growth under standard conditions and when ammonium is consecutively added to N-starved cultures. Arrows indicate time points at which 1 mM NH_4_Cl was added. Data are the means ± SD from three independent cultures (including three independent clones of each mutant). (C) Representative photographs of cultures used in the experiment. Ammonium was added after day 3 and again after days 5 and 6. (D) Whole-cell absorption spectra. Values were normalized to *A*_750_ values.

Under standard conditions, i.e., with nitrate as the sole N source and under ambient CO_2_, at which PirA is not detectable in the WT, the *pirA*-manipulated recombinant strains grew similarly to the WT, as expected (Fig. 3A and C). Given that PirA rapidly accumulated in response to increasing N availability, which suggests a function related to these conditions, it was tempting to speculate whether both recombinant strains show a phenotype, e.g., an affected pigment synthesis/degradation, when the N concentration is altered. To test this, we cultivated the WT, Δ*pirA*, and *pirA*^+^ strains under N oscillating conditions. We inoculated cultures in nitrate-free BG11 and cultivated for 3 days, which was accompanied by pigment degradation (Fig. 3B and C), causing nitrogen starvation-induced chlorosis (40). Cultures of both recombinant strains showed the same behavior as the WT and did not show a nonbleaching (*nbl*) phenotype, as is known for *nbl* mutants that are affected in phycobilisome degradation (41). Consistent with this, the phycocyanin content was strongly reduced in all cells, measured by the diminished absorption at 630 nm (Fig. 3D, day 3). The similar bleaching kinetics of all strains is consistent with the fact that PirA is not detectable under N limitation. Afterward, the fully chlorotic cells were exposed to consecutive pulses of limited amounts of ammonium (1 mM) to simulate conditions under which PirA is rapidly accumulating and likely important. The regreening process was monitored by measuring growth as well as whole-cell absorption spectra at wavelengths in a range between 400 and 750 nm. While growth recovery was rather similar in all three strains, a clearly altered pigmentation was observed in the *pirA*^+^ strain after iterated ammonium pulses (Fig. 3C and D). Consistent with the visible difference, a lower absorption at 630 nm was detected, resulting from reduced phycocyanin content. These data indicate that the cells are impeded in coping with fluctuating N concentrations and struggle to recover from chlorosis when PirA accumulation is not correctly balanced. This supports the assumption that this small protein plays a crucial role and participates in regulatory processes that control N metabolism.

### Altered PirA abundance affects metabolites of N metabolism.

**FIG 4:**
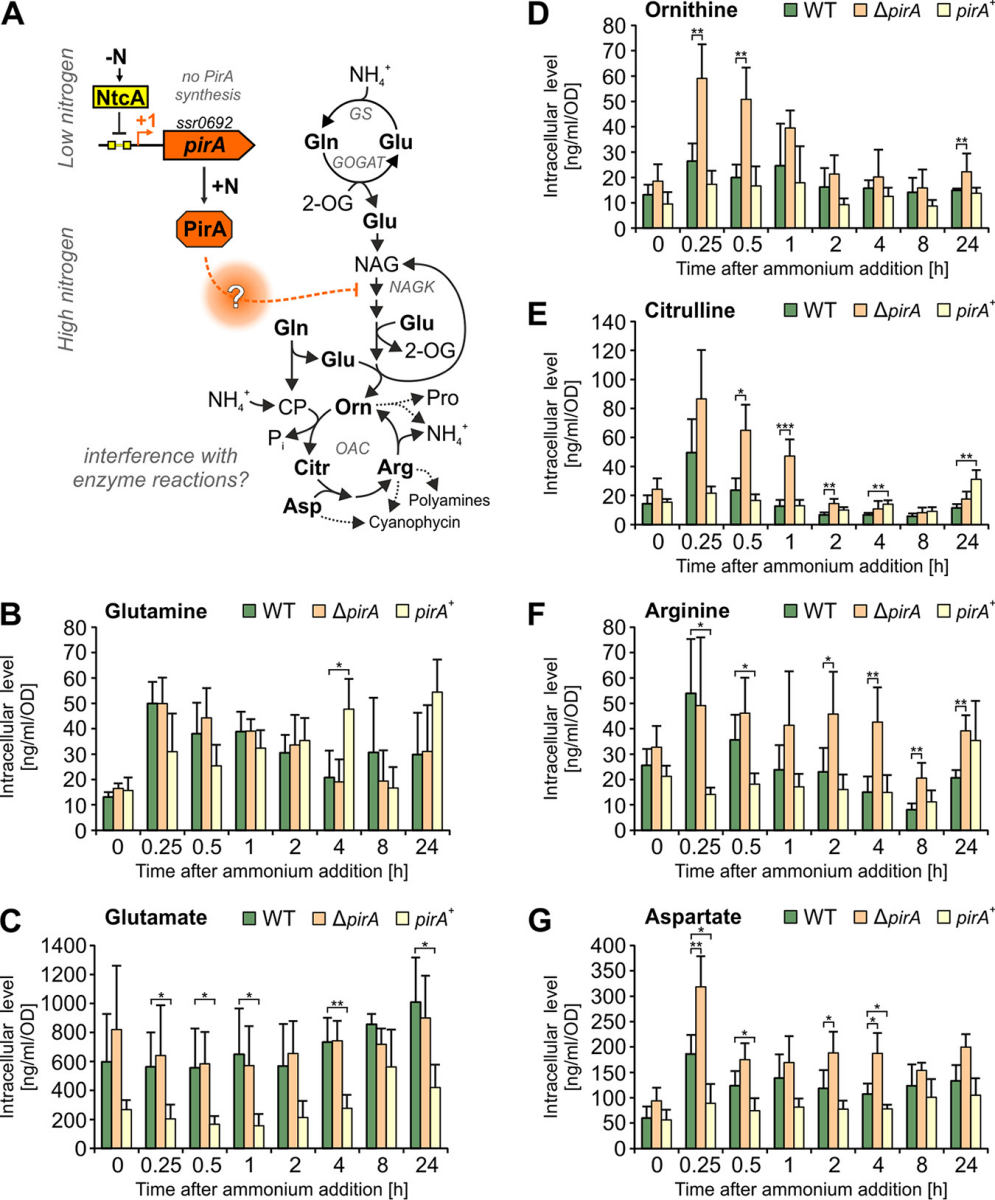
Kinetics of metabolites linked to the OAC cycle in response to ammonium addition. (A) Simplified overview of metabolic pathways associated with ammonium assimilation and a possible regulatory impact of PirA on certain enzymatic reactions. 2-OG, 2-oxoglutarate; CP, carbamoyl phosphate; GS, glutamine synthetase; GOGAT, glutamine oxoglutarate aminotransferase; NAG, N-acetyl-glutamate; NAGK, N-acetyl glutamate kinase; OAC, ornithine-ammonia cycle. (B to G) Kinetics of selected metabolites after adding 10 mM ammonium to nitrate-grown cells in the exponential phase. Metabolites were determined by ultrahigh-performance liquid chromatography-tandem mass spectrometry after ethanol extraction from cells of the WT, Δ*pirA*, and *pirA*^+^ strains. Data are the means ± SD from two independent experiments, each conducted with three biological replicates (independent clones). Significant differences in the mutant strains compared to WT at each time point are labeled and were revealed by one-way analysis of variance (ANOVA; *, *P* < 0.05; **, *P* < 0.01; ***, *P* < 0.001).

To further examine a potentially regulatory function of PirA, time-resolved quantification of selected metabolites was performed for nitrate-grown cells of the WT and both mutants after addition of 10 mM ammonium. Interestingly, perturbation of PirA levels had a distinct impact on the accumulation of several key metabolites in *Synechocystis*. Most intriguingly, the kinetics of metabolites that are part of or are associated with the recently discovered ornithine-ammonia cycle (34) were strongly affected in Δ*pirA* and *pirA*^+^ strains compared to the WT (Fig. 4; an extended data set is shown in Fig. S2). In general, N upshift triggered a transient accumulation of citrulline, ornithine, arginine, and aspartate in WT cells, similar to previous reports (34). Interestingly, the absence of PirA intensified and prolonged the accumulation of these metabolites, while the overexpression of *pirA* prevented or delayed their accumulation to a significant extent (Fig. 4). Moreover, kinetics of glutamine and glutamate, both key amino acids in N metabolism, showed striking differences between the tested strains. For instance, glutamate, which represents the main amine donor in a plethora of pathways, was significantly decreased in the *pirA*^+^ strain throughout the experiment.

10.1128/mBio.00229-21.4 FIG S2 Kinetics of further metabolites after adding 10 mM ammonium to nitrate-grown cells in the exponential phase. Metabolites were determined by UHPLC-MS/MS after ethanol extraction from cells of the WT, Δ*pirA*, and *pirA*^+^ strains. Data are the means ± SD from two independent experiments, each conducted with three biological replicates (independent clones). Significant differences in the mutant strains compared to the WT at each time point are labeled and were revealed by one-way analysis of variance (ANOVA; *, *P* < 0.05; **, *P* < 0.01; ***, *P* < 0.001). Download FIG S2, TIF file, 1.9 MB. Copyright © 2021 Bolay et al. 2021 Bolay et al. https://creativecommons.org/licenses/by/4.0/ This content is distributed under the terms of the Creative Commons Attribution 4.0 International license.

The data clearly indicate that PirA plays a pivotal role in balancing fluxes through or into key amino acids, such as arginine. In cyanobacteria, the rate-limiting step of arginine synthesis is controlled by a well-investigated regulatory mechanism through complex formation of the key enzyme NAGK with the P_II_ protein (31, 32, 42). Moreover, a P_II_ variant with highly increased affinity toward NAGK (P_II_-I86N) causes constitutive NAGK activation and, hence, arginine accumulation (43), which was at least transiently observed in cells of the Δ*pirA* strain. Thus, it was tempting to speculate that PirA interferes at this regulatory node.

### PirA interacts with the signaling protein P_II_ in an ADP-dependent manner.

**FIG 5:**
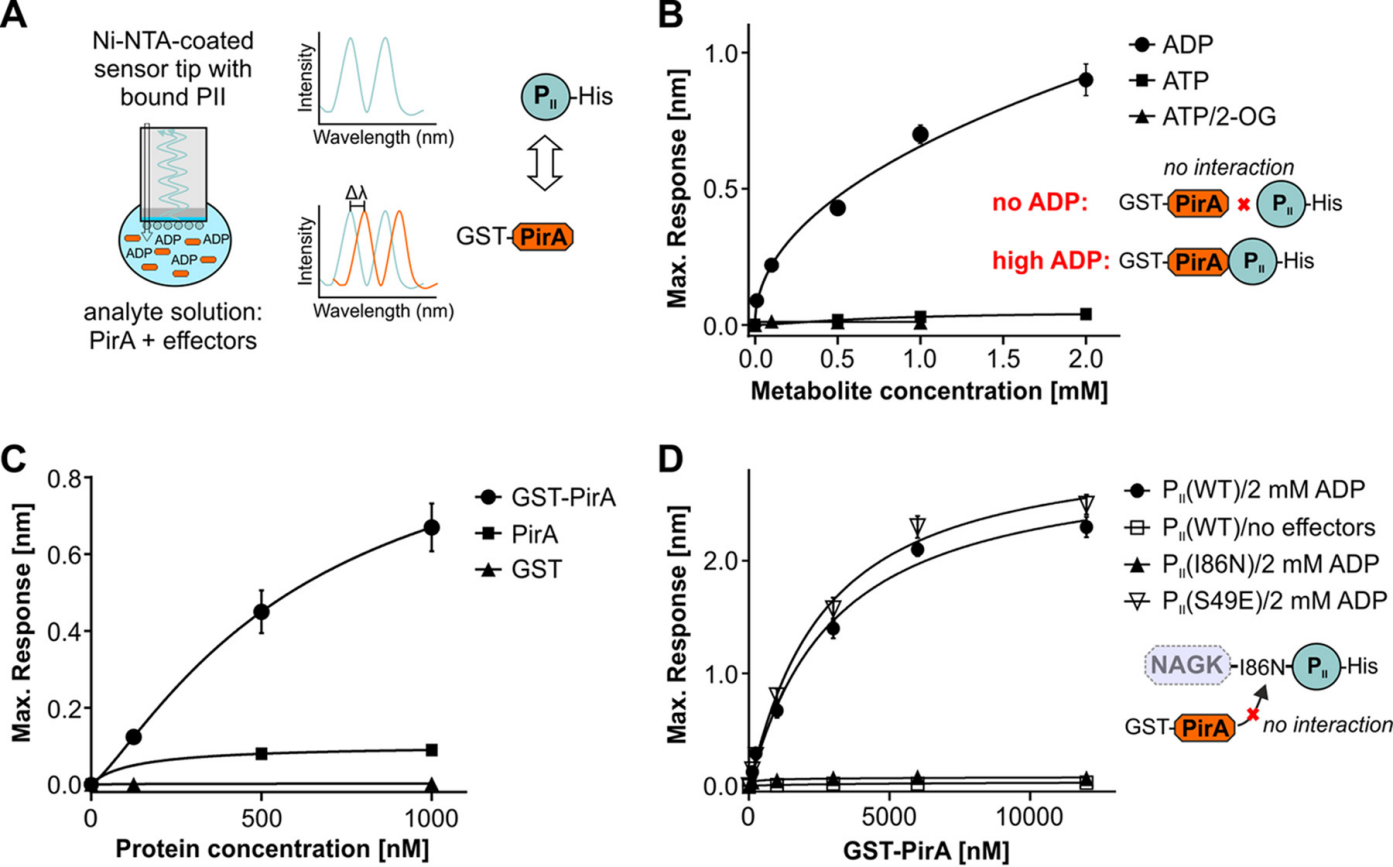
Determination of complex formation between PirA and the P_II_ protein, measured by biolayer interferometry (BLI). (A) Schematic view of the measuring principle. (B) Representation of the maximum binding response of P_II_(WT)-His and GST-PirA interaction in the presence of different concentrations of ADP, ATP, or ATP/2-OG. (C) The maximum binding response at different protein concentrations of GST-PirA, tag-free PirA, or free GST in the presence of 2 mM ADP. As the binding response is a function of the mass of bound interactor, the response with GST-tagged PirA is correspondingly higher than that with isolated PirA peptide. (D) Representation of the maximum binding response at increasing concentrations of GST-PirA in the absence of effector molecules or in the presence of 2 mM ADP with three different T-loop variants of P_II_. Data are the means ± SD from triplicate measurements.

Recently, PirA was found enriched in pulldown experiments of the signaling protein P_II_ (44). This indicated that PirA directly interacts with the P_II_ protein and thereby exercises a regulatory function similar to other small P_II_ interacting proteins, such as PipX (27) or CfrA/PirC, which has recently been discovered by two independent laboratories (45, 46). To verify the interaction between PirA and P_II_ from *Synechocystis*, *in vitro* binding experiments were performed using biolayer interferometry (BLI). To this end, recombinant protein variants were expressed in and purified from E. coli. His_8_-tagged P_II_ protein was immobilized on a nickel-nitrilotriacetic acid (Ni-NTA)-coated sensor tip, and a glutathione *S*-transferase (GST)-tagged PirA variant was used as the analyte in the presence or absence of various effector molecules (Fig. 5A). Indeed, complex formation was detected in the presence of ADP in a clear concentration-dependent manner (Fig. 5B). In contrast, no interaction was observed in the presence of ATP, mixtures of ATP and 2-oxoglutarate (2-OG), or when no effector molecule was present. These data unambiguously revealed ADP-dependent interaction between P_II_ and GST-tagged PirA. To test the specificity of the interaction, we performed similar measurements using PirA variants, where the GST tag was removed by proteolytic cleavage. The small PirA peptide yielded a binding signal that was about 6-fold lower than the signal observed for the GST fusion protein (Fig. 5C). This agrees well with the expected signal, since the BLI response depends on the mass changes at the sensor tip (GST-PirA versus PirA; 31.8 kDa/5.8 kDa = 5.5). Furthermore, BLI experiments with only the GST tag (26 kDa) did not result in any detectable signal (Fig. 5C), which clearly confirms that P_II_ specifically interacts with PirA in these binding studies. Since the GST fusion protein is easier to handle accurately and the signal is superior to that of the isolated PirA peptide, further experiments were performed with GST-tagged PirA.

To further study the P_II_-PirA interaction, different P_II_ variants were examined. In most cases, interaction of proteins with P_II_ involves the highly flexible T‐loop structure that can adopt a multitude of conformations (30, 47). Accordingly, a P_II_ variant lacking the T-loop (P_II_(ΔT)-His_8_) was also tested. As expected, no response was observed, confirming interaction with PirA via the T-loop (not shown). Moreover, we tested the variant P_II_(I86N), where a single amino acid replacement, Ile86 to Asn86, locks the T-loop in a conformation that promotes constitutive NAGK binding (48, 49). Strikingly, this variant was not able to bind PirA, even in the presence of 2 mM ADP, which otherwise promotes binding to the native P_II_ (Fig. 5D). In contrast, the phosphomimetic variant P_II_(S49E), which does not interact with NAGK (50), shows unaffected complex formation with PirA (Fig. 5D). The affinity of P_II_(S49E) to PirA was even slightly higher than that observed for the native variant [*K_D_* values, 2.9 ± 0.34 μM for P_II_(WT) and 2.5 ± 0.27 μM for P_II_(S49E)]. Obviously, a conformation of the T-loop that mediates a high P_II_ affinity to NAGK prevents its interaction with PirA. Together with the metabolite profiles showing dysregulated arginine synthesis in the Δ*pirA* mutant, the present data implicate the interference of PirA with NAGK regulation through interaction with P_II_.

### PirA antagonizes P_II_-dependent activation of arginine-inhibited NAGK.

**FIG 6:**
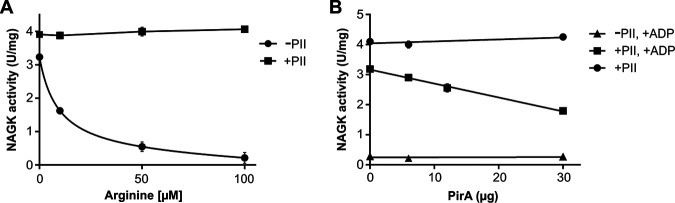
Impact of PirA on P_II_-dependent NAGK activity *in vitro*. (A) Inhibition of NAGK by arginine in the presence or absence of 2.4 μg P_II_. (B) NAGK activity as a function of increasing PirA concentration in the presence or absence of P_II_ and 1 mM ADP. The assay otherwise contained 0.1 mM arginine and 1 mM ATP. Data are the means ± SD from triplicate measurements.

To further demonstrate that PirA interferes with the P_II_-NAGK complex, an enzyme assay with purified components was conducted. Using the standard NAGK assay, where ADP formation is coupled to pyruvate-kinase and lactate-dehydrogenase activity (48), thereby keeping ADP concentrations at zero, no effect of PirA on P_II_-promoted activation of NAGK could be observed (not shown). Therefore, an assay was employed where NAG phosphorylation was coupled to subsequent NADPH-dependent N-acetyl-*γ*-glutamyl-5-phosphate reduction (51), allowing the addition of ADP. In this assay, the presence of P_II_ protects NAGK from arginine inhibition, with 100 μM arginine fully discriminating free NAGK from P_II_-complexed NAGK (Fig. 6A). When the assay was performed in the presence of 0.1 mM arginine and 1 mM ATP, again no effect of adding increasing PirA concentrations could be detected. When, however, the same assay was performed in the presence of 1 mM ATP and 1 mM ADP, a concentration-dependent inhibition of NAGK activity of up to 50% could be detected, in accord with the ADP requirement for P_II_-PirA interaction (Fig. 6B). Altogether, the data point to a PirA-mediated disaggregation of the P_II_-NAGK complex that subsequently leads to NAGK inhibition by arginine.

## DISCUSSION

Arginine serves as a proteinogenic amino acid and precursor for the synthesis of polyamines and other N storage compounds, such as the cyanobacterial cyanophycin. In bacteria, arginine can be synthesized either by a linear pathway, e.g., present in *Enterobacteriaceae*, or by an energetically more favorable cyclic pathway. In the latter, N-acetyl-ornithine reacts with glutamate to yield ornithine and N-acetyl-glutamate, the starting metabolite of the pathway, which is widely distributed in nature and also present in cyanobacteria (52, 53). Nevertheless, arginine synthesis requires vast amounts of energy and N (42) and, thus, is tightly regulated in bacteria. This is mainly achieved by feedback inhibition of the corresponding key enzymes by the end product arginine. In E. coli, this addresses N-acetylglutamate synthase (NAGS), which catalyzes the first step of linear arginine synthesis from glutamate (52). In contrast, in those bacteria harboring the cyclic pathway, the second enzyme, NAGK, is feedback inhibited by arginine (53).

### PirA, a novel player in the distinctive regulation of arginine synthesis in cyanobacteria.

In cyanobacteria, NAGK is the target of a molecular regulatory mechanism that involves complex formation with the signal transduction protein P_II_ (32, 50). This interaction diminishes feedback inhibition of NAGK by arginine and, hence, boosts the metabolic flux toward the end product (32). Its importance for the control of cyanobacterial metabolism is supported by the fact that this mechanism is widely present in oxygenic phototrophs such as plants (54, 55) and microalgae (56) but appears to be absent from other bacteria (for an overview, see reference 57). Regarding arginine metabolism, the uniqueness of cyanobacteria within prokaryotes is also exemplified by the recent discovery of active cycling between ornithine and arginine via an ornithine-ammonia cycle (OAC), similar to the known ornithine-urea cycle (OUC) that is present in terrestrial animals but typically absent from bacteria (34).

**FIG 7:**
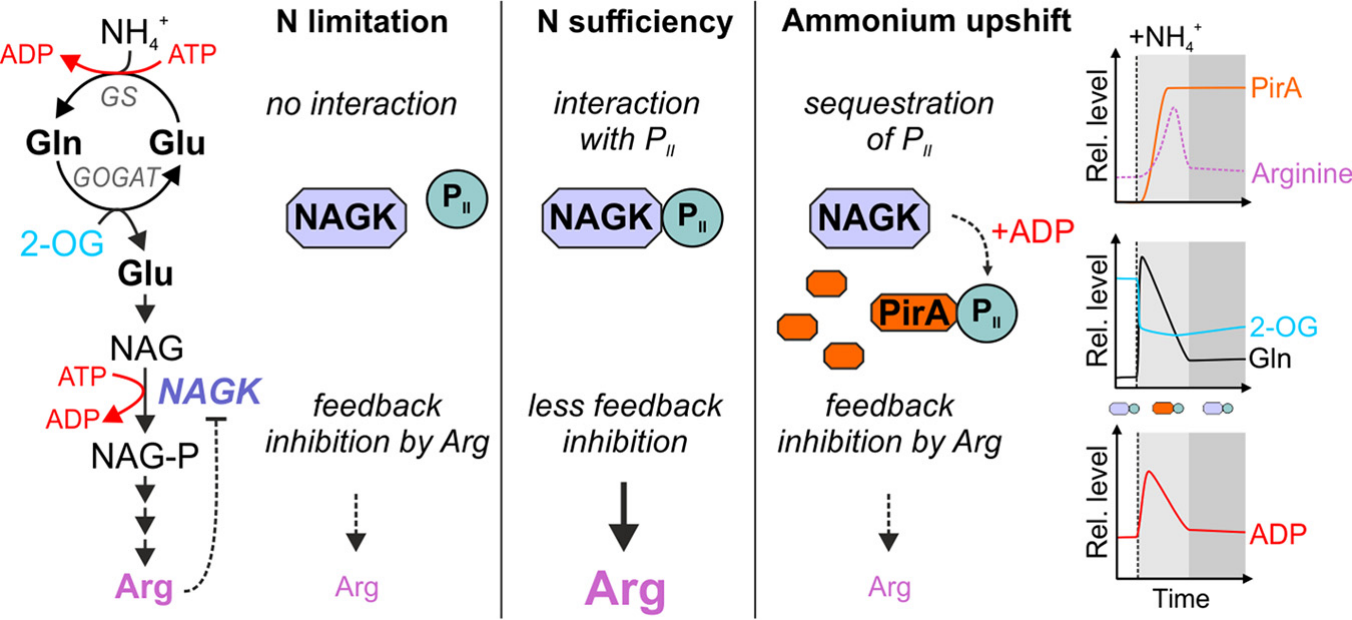
Anticipated model of PirA function. Metabolite kinetics have been approximated based on available literature data (34, 38). Upon shifts in the ammonium concentration, PirA accumulates via 2-OG-dependent derepression of the *pirA* gene. The gene product is presumably required to slow down ATP-consuming synthesis of arginine. This could be achieved by ADP-dependent sequestration of P_II_ protein bound to NAGK, which is required to diminish feedback inhibition of the enzyme and, in turn, activate arginine synthesis. The sequestration of P_II_ results in stronger arginine feedback inhibition of NAGK, diminishing energy consumption and flux into arginine. After metabolic reorganization (e.g., by inactivating glutamine synthetase activity and decreasing ATP consumption), ADP levels may fall below a critical level, preventing interaction between PirA and P_II_. Accordingly, a higher fraction of the P_II_ pool will again interact with and activate NAGK, which in turn results in elevated arginine synthesis.

Here, we introduce the small cyanobacterial protein PirA as a novel key regulator in the cyanobacterial arginine synthesis pathway and, hence, the OAC. Our data confirm PirA accumulation under N excess, particularly when ammonium is added. This accumulation is obviously required to adjust a certain pool of metabolites that are part of the OAC, including arginine. Mechanistically, we propose a model where PirA competes with NAGK for the P_II_ protein (Fig. 7). In response to ammonium addition, the accumulating PirA molecules interfere with the complex formation between P_II_ and NAGK, thereby mitigating NAGK activation and preventing an overaccumulation of arginine.

Surprisingly, detectable PirA accumulation only occurred under ammonium shock, even when *pirA* mRNA was transcribed independently from N and energy status of the cell. A similar situation can be encountered with the GS IFs, which were shown to be degraded by metalloproteases when not bound to GS (37). Thus, it is tempting to speculate that PirA accumulation can only occur when bound to P_II_. However, to reveal the mechanism of PirA turnover, detailed research beyond the scope of this study is required.

### PirA integrates N and energy sensing.

Remarkably, PirA forms a complex with P_II_ only in the presence of ADP. Accordingly, the pivotal stimulus for the suggested regulatory mechanism is not only the N status, which is mainly sensed by 2-OG in cyanobacteria, similar to other prokaryotes (58, 59). The 2-OG level determines the affinity of NtcA to its binding motif (24, 29), which therefore also determines PirA accumulation. Moreover, PirA was found to transiently but strongly accumulate during low C supply (60). This is consistent with the derepression of the *pirA* gene by the dissociation of NtcA from its binding motif upstream, since low C supply, i.e., a low C/N ratio, leads to a decreased 2-OG pool (61). The interaction between PirA and P_II_ strongly responds to ADP as another signal and, hence, depends on the cellular energy status. These observations resemble another small P_II_-interacting protein, PipX, whose interaction with P_II_ is enhanced by ADP as well (51, 62). PipX functions as a coactivator of the transcription factor NtcA and is required for the 2-OG-dependent DNA binding and transcriptional activation of genes (27). In the presence of high ADP levels, PipX preferably interacts with P_II_, which attenuates NtcA activity and in turn leads to derepression and upregulation of the *pirA* gene, similar to the *gif* genes encoding the GS IFs (33).

Interestingly, *in vitro* data showed that the NAGK-P_II_ interaction is also ADP sensitive. While ADP does not entirely prevent complex formation, it increases dissociation of NAGK from ADP-bound P_II_ (48). However, detailed analysis revealed that increasing ADP levels have only a minor effect on the NAGK-P_II_ complex as long as ATP is present, whereas this interaction is mainly tuned by changing the 2-OG levels (51, 63). It should be kept in mind that the sensing properties of P_II_ are influenced by the binding partner in such a way that, for certain targets, small fluctuations in the ADP/ATP ratios are sensed (e.g., P_II_-PipX complex formation), whereas in other cases fluctuations in the 2-OG levels are perceived (e.g., P_II_-NAGK interaction). In light of these data, PirA appears to amplify the energy-dependent signal in P_II_-mediated activation of NAGK. By sequestering P_II_ under conditions of low 2-OG and high ADP levels, PirA may shift the equilibrium of the P_II_-NAGK complex toward noncomplexed NAGK, i.e., in case the cell transiently experiences energy limitation. Given that bacteria employ GS-GOGAT only during sufficient energy supply (64), it can be assumed that the high ATP consumption by GS in consequence of ammonium addition to nitrate-grown cells could create such an energy limitation. This, in turn, might require the limiting of ATP-dependent flux via NAGK into arginine and to couple such a regulatory mechanism to the intracellular ADP levels, which are a direct result of high ATP turnover.

Depending on the detailed structure of the complexes, P_II_ phosphorylation either abrogates interaction with its targets, as demonstrated for NAGK (32), or has no effect on them, as in the case of P_II_-PipX interaction (35). The phosphomimetic variant P_II_(S49E) showed slightly enhanced interaction with PirA compared to P_II_(WT). In a similar way, P_II_ interacts with PipX irrespective of Ser49 modification; nevertheless, the interaction is T-loop dependent (27). The major interaction surface is at the proximal part of the T-loop, whereas the tip region with the critical Ser49 residue does not participate in complex formation. From the Ser49-independent mode of PirA-P_II_ interaction, we conclude that the PirA-P_II_ interaction also takes place with phosphorylated P_II_ that does not bind to NAGK. In any case, the interaction of P_II_ with NAGK or PirA appears to be mutually exclusive, since both P_II_ interaction partners require the T-loop in different conformations for interaction. This explains the effect of PirA on the *in vitro* activity of the P_II_-NAGK complex in the presence of a concentration of arginine that is inhibitory for free NAGK but permissive for the P_II_-NAGK complex. By competing for P_II_ binding, PirA releases free NAGK, which is strongly subject to arginine feedback inhibition. The efficiency of competition between NAGK and PirA depends on the cellular ATP/ADP ratio.

The competition between NAGK and PirA for P_II_ is further illustrated by the inability of PirA to bind P_II_(I86N). This variant adopts a constitutive NAGK-bound-like structure of the T-loop (48, 49). Accordingly, the lack of PirA interaction with P_II_(I86N) also agrees with the strong *in vivo* activation of NAGK by this variant (43). However, for a more detailed understanding of the mechanism, by which PirA affects P_II_ signaling, further functional biochemical studies and structural analyses are required.

Homologs of *pirA* are widespread in cyanobacterial genomes, which generally supports its crucial regulatory role. For instance, the *pirA* homolog *asr1328* of the filamentous, diazotrophic cyanobacterium *Anabaena* sp. strain PCC 7120 was shown to be negatively regulated by NtcA as well (65). Similar to the GS IFs, gene annotations in a few strains, such as Thermosynechococcus elongatus or *Synechococcus* sp. strain JA-3-3Ab, suggest that alternative versions of PirA with an extended N terminus also exist (see Fig. S3 in the supplemental material). Nevertheless, the existence of those proteins has not been experimentally investigated yet; hence, false annotations cannot be excluded. This assumption is supported by the fact that only a few of those elongated sequences could be found in protein databases and show only partial similarity. Therefore, the function of these PirA-like proteins with N-terminal extensions remains elusive. In addition, it is worth noting that PirA is, similar to the GS IFs, completely absent from marine picocyanobacteria (Fig. 1). In accordance with this, this clade lacks several salient features of N-sensing and utilization that are widespread among cyanobacteria. For instance, P_II_ is not subject to phosphorylation in *Prochlorococcus* (66, 67), and both *Prochlorococcus* and *Synechococcus* genera are incapable of cyanophycin synthesis and lack several OAC cycle genes (68). These genome-streamlined strains occur in oligotrophic realms of the ocean with hardly any fluctuation in nutrient supply (69). Thus, it is compelling to speculate that PirA-mediated short-term adjustment of arginine synthesis to the N and energy status of the cell is not required in such habitats.

10.1128/mBio.00229-21.5 FIG S3 Alternative PirA variants. BLASTP analyses suggest alternative PirA versions with an extended N-terminal end in a few strains. This resembles the glutamine synthetase (GS) inactivating factors (IF), all of which show a homologous C terminus but only some show an N-terminal extension. These extensions show conserved amino acid residues that confer stability to the proteins that carry it (for further information, see Saelices et al., Mol. Microbiol. 82:964–975, 2011). Those residues are also present in the N-terminal part of alternative PirA versions. Download FIG S3, TIF file, 1.0 MB. Copyright © 2021 Bolay et al. 2021 Bolay et al. https://creativecommons.org/licenses/by/4.0/ This content is distributed under the terms of the Creative Commons Attribution 4.0 International license.

## MATERIALS AND METHODS

### Strains and growth conditions.

*Synechocystis* sp. strain PCC 6803, originally obtained from N. Murata (Japan), was used as the wild type. Cells were grown in BG11 medium (70) depleted of Cu^2+^ ions and supplemented with 10 mM TES buffered at pH 8.0. The sole N source in that medium is nitrate at a concentration of 17.64 mM (termed nitrate grown). Cultivation was performed in baffled Erlenmeyer flasks in the presence of ambient CO_2_ under constant illumination (white light, 50 μmol photons m^−2^ s^−1^) at 30°C, 75% humidity and 150 rpm. Each recombinant strain was isolated on BG11 agar plates and maintained in medium containing either kanamycin or gentamicin at a concentration of 50 μg/ml and 2 μg/ml, respectively. Prior to the experiments investigating the impact of altered PirA abundance, the cultures were supplemented with 1 μM CuSO_4_.

### Mutant strain generation.

To knock out the *ssr0692* (*pirA*) gene, the upstream and downstream regions of *pirA* were amplified from *Synechocystis* genomic DNA (gDNA) using primer combinations Ssr0692upst_fw/Ssr0692upst_rev and Ssr0692downst_fw/Ssr0692downst_rev (all primers are listed in Table S1 in the supplemental material). The kanamycin resistance cassette was amplified from a customized construct obtained by gene synthesis using primers KmR_fw and KmR_rev. The synthesized construct harbored the *aphII* gene and its promoter, which were originally obtained from pUC4K (Amersham). In addition, an *oop* terminator was introduced downstream of *aphII*. All amplicons had short fragments of sequence complementarity and were fused via polymerase cycling assembly (PCA) using primers Ssr0692upst_fw/Ssr0692downst_rev. The resulting construct was introduced into pJET1.2 (Thermo Scientific) and used to transform chemically competent E. coli DH5α. After isolation of the pJET_ssr0692_KmR_KO plasmid from E. coli, the knockout construct was introduced into *Synechocystis* WT by natural transformation and homologous recombination into the chromosome. To enable the overexpression of *pirA*, the 5′ untranslated region (UTR) and 3′UTR of the *petE* gene were amplified from *Synechocystis* gDNA using primers P*petE*_fw(XhoI)/5′*petE*_ssr0692_rev and 3′*petE*_ssr0692_fw/Toop_rev(AseI). The *pirA* coding sequence was amplified from *Synechocystis* gDNA using primers ssr0692_fw/rev. All amplicons had short fragments of sequence complementarity and were fused via polymerase cycling assembly (PCA) using primers P*petE*_fw(XhoI)/Toop_rev(AseI) and introduced into the broad-host-range plasmid pVZ322 via restriction digestion and ligation into XhoI/AseI sites. The recombinant plasmid pVZ322-P*petE*:*pirA*, obtained after transformation of and purification from E. coli DH5α, was introduced into *Synechocystis* WT via electroporation. All strains and constructs were verified by PCR and Sanger sequencing.

10.1128/mBio.00229-21.1 TABLE S1 Primers used in this study. Download Table S1, DOCX file, 0.01 MB. Copyright © 2021 Bolay et al. 2021 Bolay et al. https://creativecommons.org/licenses/by/4.0/ This content is distributed under the terms of the Creative Commons Attribution 4.0 International license.

To generate the alternative *ΔpirA*/P*_petE_-pirA* strain (chromosomal integration), a DNA fragment containing the *pirA* open reading frame and the coding sequence for six histidine residues at the carboxyl-terminal end was amplified by PCR using *Synechocystis* genomic DNA and primers ssr0692.KpnI and ssr0692.HisBamHI. This fragment was cloned into KpnI-BamHI-digested pPLAT plasmid (37), a pGEM-T derivative containing a 2-kb region of the nonessential *nrsBACD* operon, which includes targets for these restriction enzymes (71). This cloning generated pPLAT-*pirA*. In a second step, the *Synechocystis petE* promoter was amplified by PCR using genomic DNA and primers P*petE*.KpnI.1 and P*petE*.KpnI.2 and cloned into the KpnI site of pPLAT-*pirA*. Finally, a Km^r^ CK1 cassette from pRL161 (72) was cloned in the BamHI site of pPLAT-*pirA*, generating pPLAT-P*petE-pirA*. For the amplification of the plasmids, chemically competent E. coli DH5α cells were used in all cases. pPLAT-P*petE-pirA* plasmid was used to transform a *Synechocystis ΔpirA* strain. All generated strains and their properties are given in Table S2.

10.1128/mBio.00229-21.2 TABLE S2 Strains used in this study. Download Table S2, DOCX file, 0.01 MB. Copyright © 2021 Bolay et al. 2021 Bolay et al. https://creativecommons.org/licenses/by/4.0/ This content is distributed under the terms of the Creative Commons Attribution 4.0 International license.

### N oscillation experiment.

WT and mutant strains were inoculated in triplicates at an optical density at 750 nm (OD_750_) of 0.1 in 3-baffled 100-ml flasks in 20 ml N-depleted BG11 supplemented with 1 μM CuSO_4_. For comparison, the same strains were also cultivated in standard BG11 medium containing 17.64 mM nitrate. After 3 days, the N-starved cells were supplemented with 1 mM NH_4_Cl, a step that was repeated after 5 and 6 days. Whole-cell spectra of cell suspensions were conducted with a Cary 300 UV-visible spectrophotometer (Agilent).

### RNA extraction and Northern blots.

Cells for Northern blot analysis were harvested by rapid filtration on polyether sulfone filters (pore size 0.8 μm; Pall). Filters were immediately resolved in 1 ml PGTX solution (73) and frozen in liquid N_2_. RNA extraction was conducted as previously described (74). For Northern blots, 3 μg of RNA was separated on 1.5% agarose gels supplemented with 6% formaldehyde. The gels were run in MEN buffer containing 20 mM MOPS, 5 mM NaOAc, 1 mM EDTA, pH 7.0. Prior to loading, the RNA samples were incubated at 65°C for 10 min in loading buffer containing a final concentration of 62.5% (vol/vol) deionized formamide (Sigma-Aldrich). Afterward, RNA was transferred via capillary blotting to an Amersham Hybond N^+^ nylon membrane (GE Healthcare) and cross-linked at 1,250 μJ in a UVP cross-linker (Analytik Jena). To specifically detect the *pirA* transcript, the RNA-mounted nylon membrane was hybridized with a complementary α-^32^P-labeled single-stranded RNA (ssRNA) probe that was generated by *in vitro* transcription using the MAXIscript T7 transcription kit (Thermo Fisher Scientific). As a transcription template, a DNA fragment obtained by PCR with primers Ssr0692_T7_fw and Ssr0692_rev was used. Subsequently, Fuji BAS-IIIS imaging plates were exposed to the membranes and read out by an Amersham Typhoon laser scanner (GE Healthcare). As a loading control, the same membranes were hybridized with ssRNA probes complementary to the 5S rRNA, which were generated in the same way using primers 5sRNA_fw/rev (Table S1).

### Anti-PirA antibody production.

A DNA fragment encompassing the *pirA* open reading frame (ORF) was amplified by PCR from *Synechocystis* genomic DNA, using the oligonucleotides Ssr0692ORF-fw and Ssr0692ORF-rv. This fragment was cloned into NdeI-XhoI-digested pET24a(+) plasmid (Novagen) to generate pET24-Ssr0692 plasmid. Exponentially growing E. coli BL21 cells transformed with pET24-Ssr0692 were treated with 1 mM isopropyl ß-d-1-thiogalactopyranoside for 4 h. For purification of PirA-His_6_ protein, cells were collected, resuspended in buffer A (20 mM sodium phosphate, pH 7.5, 150 mM NaCl, 5 mM imidazole) with 1 mM phenylmethylsulfonyl fluoride (PMSF), and disrupted by sonication. The lysate was centrifuged at 18,000 × *g* for 20 min. PirA-His_6_ from the supernatant was purified by Ni-affinity chromatography using a HisTrapHP column (GE Healthcare) and following the manufacturer’s instructions. Elution was performed with a linear gradient (5 to 500 mM imidazole) in buffer A. Fractions with PirA-His_6_ were pooled, concentrated using centrifugal filter units (Amicon Ultra-15 3 kDa) (Millipore), and subjected to gel filtration chromatography using a Hiload 16/60 Superdex 75 gel filtration column (GE Healthcare) running on an AKTA fast protein liquid chromatography (FPLC) system and using buffer A without imidazole. Fractions containing purified PirA-His_6_ protein were pooled, concentrated, and quantified in a NanoDrop 1000 spectrophotometer (Thermo Scientific) using the extinction coefficient of PirA-His_6_ calculated with the ExPASy-ProtParam tool. Anti-PirA antiserum was obtained according to standard immunization protocols. A female specific-pathogen-free rabbit was used. Before immunization, preimmune serum was obtained to carry out the pertinent controls. Three immunizations were carried out with the antigen, PirA protein, separated by a period of 2 weeks. For the first immunization, 1.5 mg of PirA was used, and for the next two immunizations, 1 mg of antigen was used. In all cases, 0.5 ml of antigen was emulsified with 0.5 ml of Freund's incomplete adjuvant, and 3 to 5 subcutaneous injections were made. Ten days after the last immunization, the animal was exsanguinated and the serum obtained was stored at −80°C.

### Preparation of crude extracts and Western blot analysis.

For the analysis of protein abundance, cells of 2 ml culture were harvested and resuspended in 80 μl of 50 mM HEPES-NaOH buffer (pH 7.0), 50 mM KCl, 1 mM PMSF. Crude extracts were prepared using glass beads as previously described (75). For Western blot analysis, proteins were fractionated on 15% SDS-PAGE (76) and transferred to nitrocellulose membranes (Bio-Rad). Blots were blocked with 5% (wt/vol) nonfat dry milk (AppliChem) in phosphate-buffered saline (PBS)-Tween 20 for 1 h. Antisera were used at the following dilutions: anti-PirA (1:5,000) and anti-TrxA (1:10,000) (77). In all cases, the incubation of the membranes with the primary antibody was carried out overnight at 4°C. After four washes (15 min for each one) with PBS-Tween 20, the nitrocellulose membranes were incubated with a secondary antibody against rabbit IgG (1:25,000) (Sigma-Aldrich) for 1 h at room temperature. After washing again four times with PBS-Tween 20, the ECL Prime Western blotting detection reagent (GE Healthcare) was used to detect the different antigens by following the manufacturer's instructions.

### Metabolite analysis.

For metabolite analysis, cells were grown in BG11 containing the standard nitrate amount until reaching an OD_750_ of ∼0.8. Cells were harvested shortly before and after the addition of 10 mM ammonium chloride by centrifugation of 2 ml culture at 17,000 × *g* for 1 min. Supernatant was discarded and pellets were snap-frozen in liquid N. Metabolite extraction was performed by resuspending cell pellets in 1 ml of 80% (vol/vol) ethanol supplemented with 1 μg/ml l-carnitine hydrochloride as an internal standard and heating for 2 h at 60°C. After centrifugation at 17,000 × *g* for 5 min, the supernatant was transferred to a fresh vial and the pellet was again resuspended in 1 ml of 80% (vol/vol) ethanol and heated at 60°C for 2 h. After centrifugation at 17,000 × *g* for 5 min, supernatants were combined and dried in a centrifugal evaporator (Concentrator plus; Eppendorf, Germany).

The dried extracts next were dissolved in 1,000 μl liquid chromatography-mass spectrometry (LC-MS)-grade water and filtered through 0.2-μm filters (Omnifix-F; Braun, Germany). A volume of 1 μl of the cleared supernatants was analyzed using the high-performance liquid chromatograph mass spectrometer LCMS-8050 system (Shimadzu) and the incorporated LC-MS/MS method package for primary metabolites (version 2; Shimadzu) as described previously (78). Briefly, quantification of metabolites was done via multiple reaction monitoring (MRM; positive ion mode) based on the specific mass/charge ratio (*m/z*) values of the analyzed compounds defined in the method package: alanine, 89.90→44.10; arginine, 175.10→70.10 and 60.10; asparagine, 133.10→87.15 and 28.05; aspartate, 134.11→88.10 and 74.05; carnitine, 162.10→103.05 and 60.10; citrate, 191.20→111.10 and 87.05; citrulline, 176.10→159.00 and 70.05; glutamate, 147.90→84.10 and 56.10; glutamine, 147.14→130.10 and 84.15; histidine, 155.90→110.10 and 56.10; isoleucine, 132.10→86.20 and 69.15; leucine, 132.10→86.05 and 30.05; ornithine, 133.10→116.00 and 70.10; phenylalanine, 166.10→120.10 and 103.10; proline, 116.10→70.15 and 28.00; serine, 105.90→60.10; succinate, 117.30→73.00 and 99.05; threonine, 120.10→74.15 and 56.05; tryptophan, 205.10→188.10 and 146.10; tyrosine, 182.10→130.10 and 146.10; valine, 118.10→72.15 and 91.00; 3PGA, 185.10→97.10 and 79.10. Authentic standard substances (Merck) of each compound at various concentrations were used for verification and quantification. Peak areas were normalized to signals of the internal standard (carnitine).

### BLI.

Protein interaction studies via BLI were performed on an Octet K2 instrument (Fortébio Molecular Devices [UK] Limited, Wokingham, United Kingdom), which allows simultaneous binding experiments on two channels, one of which is used as a negative control. All experiments were done in buffer containing 50 mM Tris-HCl, pH 7.4, 150 mM KCl, 2 mM MgCl_2_, 0.02% lauryldimethylamine oxide, and 0.2 mg/ml bovine serum albumin. Effector molecules were used at the following concentrations: 2 mM ATP, 2 mM 2-OG, and 0.1, 0.25, 0.5, 1, and 2 mM ADP. Interaction experiments were performed as reported previously (46). Briefly, His_8_-tagged variants of P_II_, namely, P_II_(WT), P_II_(S49E), P_II_(I86N), and P_II_(ΔT)-His_8_, were used as ligands bound to Ni-NTA-coated sensor tips. Various concentrations of GST-PirA, from 125 to 12,000 nM, were used as analytes to display association reactions at 30°C. As preliminary experiments showed GST-PirA binds unspecifically to the Ni-NTA sensor tips, the noninteracting P_II_(ΔT) variant was used to saturate the tips and thereby remove unspecific binding. The binding of P_II_ ligands was performed by first loading 10 μg/ml P_II_ on the Ni-NTA sensor tip, followed by dipping the tip into buffer for 60 s (to remove unbound P_II_) and recording the first baseline. To block unoccupied sites on the sensor surface that cause disturbing unspecific binding, 72 μg/ml P_II_(ΔT) was loaded onto the tip. Afterward, a second baseline was recorded for 60 s. Association and dissociation of the analyte were carried out by dipping the tip first into GST-PirA solution for 180s and then transferring it into buffer solution for a further 120s. In every single experiment, one sensor loaded with P_II_(ΔT) was used as a negative control. To investigate the binding of GST tag alone and PirA without the GST-tag to P_II_ protein, parallel experiments were performed in the presence of 2 mM ADP. The interaction curves were achieved by subtracting the control curve and adjusting them to the average of baseline and dissociation steps. In every set of experiments, *K_D_* values were calculated by plotting concentration versus maximum response.

His_8_-tagged variants of P_II_ were prepared as previously described (79). For the preparation of recombinant PirA protein for BLI analysis, the *pirA* gene was cloned into XhoI and EcoRI sites of pGEX-4T-3 vector (GE Healthcare Life Sciences, Freiburg, Germany), encoding recombinant PirA with N-terminally fused GST tag. In addition, *pirA* was cloned into the SapI site of pBXC3GH vector (Addgene, Toddington, UK), encoding PirA with a C-terminally fused GFP-His_10_ tag. The plasmids were overexpressed in E. coli strain BL21(DE3). Purification of PirA with GST or GFP-His_10_ tags was performed as previously described (80, 81). To remove the GFP-His_10_ tag from PirA, 2.4 mg of recombinant PirA in 750 μl of PirA buffer (50 mM Tris-HCl, 100 mM KCl, 100 mM NaCl, 5 mM MgCl_2_, 0.5 mM EDTA, 1 mM dithiothreitol, pH 7.8) was treated with 50 μl 3C protease (0.1 mg) at 4°C overnight. Afterward, 200 μl Ni-NTA agarose beads (Qiagen GmbH, Hilden, Germany) were added to the mixture and gently shaken at room temperature for 60 min. The beads were removed by filtration, and the tag-free PirA protein was dialyzed against PirA buffer containing 50% (vol/vol) glycerol and stored at −20°C until use.

### NAGK activity measurements.

To study the effect of PirA on the activity of the P_II_-NAGK complex, the activity of a recombinant NAGK from *Synechocystis* was assayed in the presence of PirA by coupling NAGK-dependent NAG phosphorylation to an auxiliary enzyme, the N-acetyl-*γ*-glutamyl-5-phosphate reductase (AGPR) from E. coli, which catalyzes the reduction of NAG-phosphate using NADPH as the reductant. The change in NADPH absorbance was recorded at 340 nm as described previously (51, 82). The reaction buffer contained 50 mM imidazole (pH 7.5), 50 mM KCl, 20 mM MgCl_2_, 0.2 mM NADPH, 0.5 mM DTT, 1 mM ATP, 1 mM ADP, and 0.1 mM arginine. Each reaction consisted of 10 μg of AGPR, 6 μg NAGK, and 2.4 μg P_II_. A constant concentration of 3 mM NAG and different concentrations of PirA from 0 to 30 μg were applied. The reaction was started by the addition of NAGK, and the absorbance was recorded over a period of 10 min with a spectrophotometer (SPECORD 200; Analytik Jena). The velocity of the reaction was calculated with a molar absorption of 1 NADPH of Σ_340_= 6,178 liters mol^−1 ^cm^−1^ from the slope of the change of absorbance per time.
