# Supplementary figures and images for: The Novel PII-Interacting Protein PirA Controls Flux into the Cyanobacterial Ornithine-Ammonia Cycle
Source: mBio. 2021 Mar 23;12(2):e00229-21. doi: 10.1128/mBio.00229-21 (PMC8092223; doi:10.1128/mBio.00229-21)

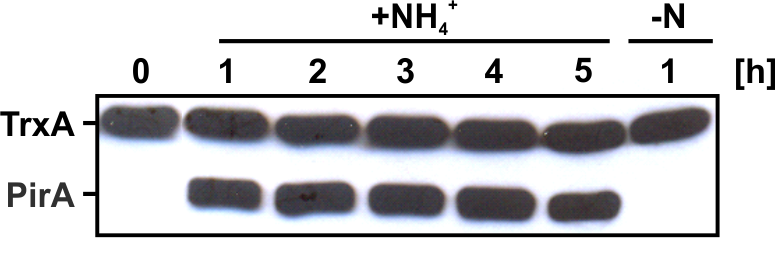

Supplement: FIG S1 [file mBio.00229-21-sf001.tif]

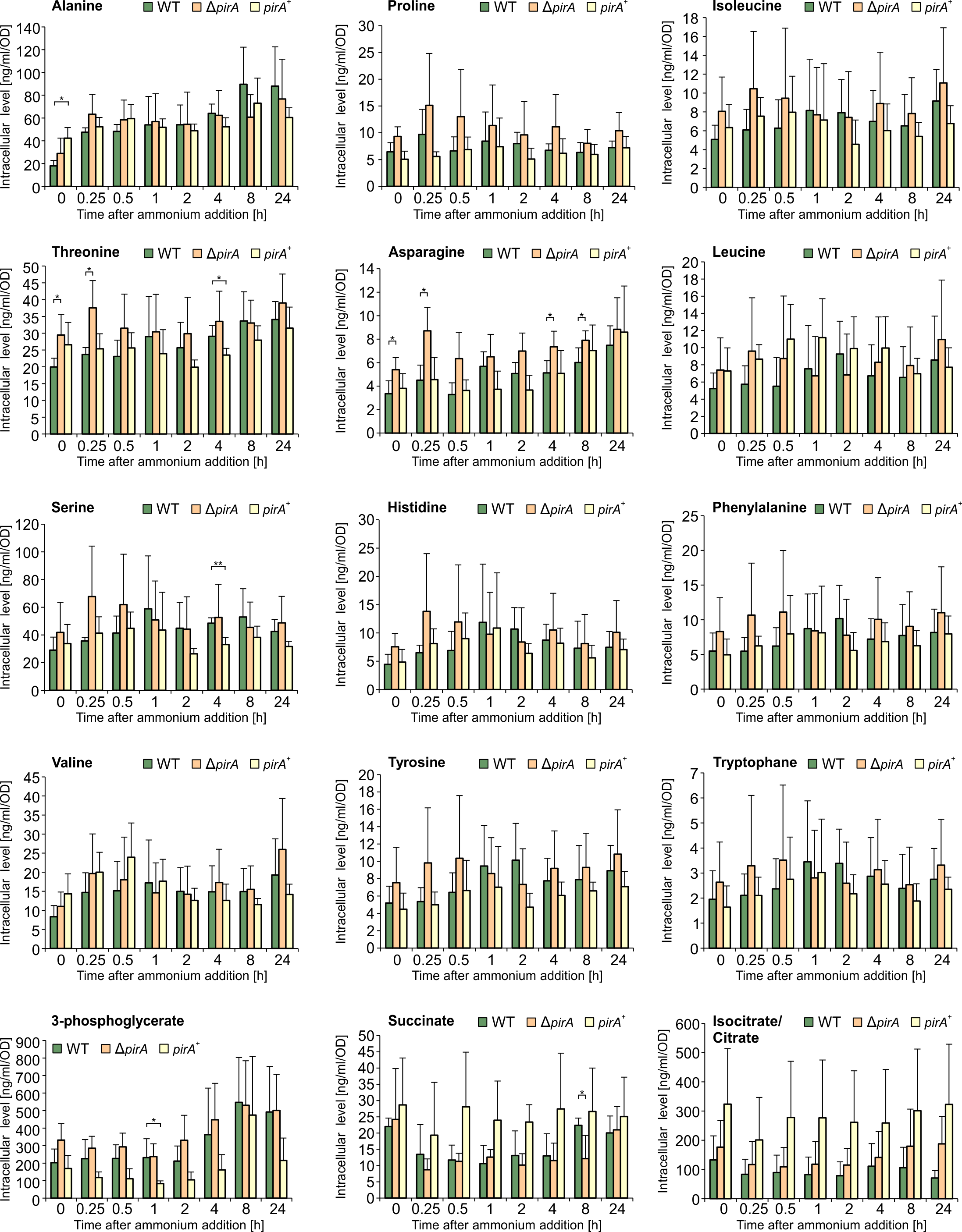

Supplement: FIG S2 [file mBio.00229-21-sf002.tif]

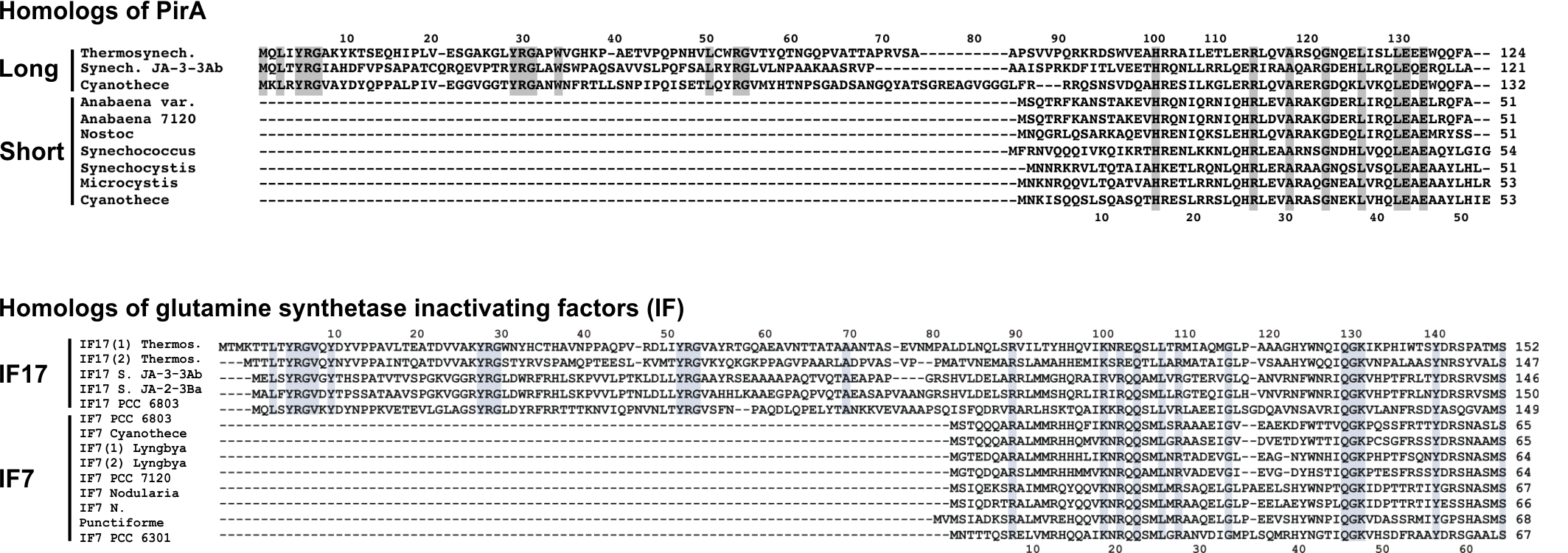

Supplement: FIG S3 [file mBio.00229-21-sf003.tif]
