# Supplementary material for: The Novel PII-Interacting Protein PirA Controls Flux into the Cyanobacterial Ornithine-Ammonia Cycle
Source: mBio. 2021 Mar 23;12(2):e00229-21. doi: 10.1128/mBio.00229-21 (PMC8092223; doi:10.1128/mBio.00229-21)
Supplement: TABLE S1 [file mBio.00229-21-st001.docx]

**Supplementary Table S1: Primers used in this study.**

| **Designation** | **5‘-3‘ sequence** | **Used for** |
| --- | --- | --- |
| 5’*petE*-ssr0692_rev | gattattcatacttcttggcgattgtatct | Amplification of *petE* 5’UTR |
| P*petE*_fw(XhoI) | actcgaggaagggatagcaagc | Amplification of *petE* 5’UTR |
| 3’*petE*-ssr0692_fw | cacctgtaatcagccagctcaatct | Amplification of *petE* 3’UTR |
| Toop_rev(AseI) | gattaataataaaaaacgcccggcgg | Amplification of *petE* 3’UTR |
| ssr0692_fw | ccaagaagtatgaataatcgtaaacgtgttttga | Amplification of *ssr0692* |
| ssr0692_rev | gctggctgattacaggtggagataagca | Amplification of *ssr0692* |
| Ssr0692-Probe_T7_fw | taatacgactcactatagggctaaggactgattgccggcg | Probe template generation for northern blot specific for *ssr0692* |
| Ssr0692-Probe_rev | gaataatcgtaaacgtgttttgactcaaac | Probe template generation for northern blot specific for *ssr0692* |
| Ssr0692upst_fw | tcagcaagatagagtttccacttcggt | Amplification of *ssr0692* upstream region |
| Ssr0692upst_rev | cggccgcgtttccacaagaataagctcaa | Amplification of *ssr0692* upstream region |
| KmR_fw | ttgtggaaacgcggccgcag | Amplification of Kanamycin resistance casette |
| KmR_rev | acaatagataaataaaaaacgcccggc | Amplification of Kanamycin resistance casette |
| Ssr0692dwnst_fw | gttttttatttatctattgttactgaagttaacaaaaatgt | Amplification of *ssr0692* downstream region |
| Ssr0692dwnst_rev | ctagaaagattctggggggaagg | Amplification of *ssr0692* downstream region |
| Ssr0692_KO-seg_fw | tcagaccgaagtggaaact | Segregation primer for *ssr0692* knockout verification |
| Ssr0692_KO-seg_rev | gtacttttcaagcggcca | Segregation primer for *ssr0692* knockout verification |
| 5sRNA_for | taatacgactcactataggagaaagaggaacttggcatcggac | Probe template generation for northern blot specific for 5s rRNA |
| 5sRNA_rev | gtcatggaaccactccgatccc | Probe template generation for northern blot specific for 5s rRNA |
| Ssr0692ORF-fw | gctactaatgaataatcgtaaacgtg | Amplification of *ssr0692* |
| Ssr0692ORF-rv | gctactcgaggtggagataagcagcttc | Amplification of *ssr0692* |
| ssr0692.KpnI | gctggtaccatgaataatcgtaaacgtg | Amplification of *ssr0692* |
| ssr0692.HisBamHI | gctggatccttagtggtggtggtggtggtgcaggtggagataagcagc | Amplification of *ssr0692* |
| P*petE*.KpnI.1 | gctggtaccctcagggagcgacttcagc | Amplification of P*petE* |
| P*petE*.KpnI.2. | gctggtaccacttcttggcgattgtatc | Amplification of P*petE* |
