# Supplementary material for: The Novel PII-Interacting Protein PirA Controls Flux into the Cyanobacterial Ornithine-Ammonia Cycle
Source: mBio. 2021 Mar 23;12(2):e00229-21. doi: 10.1128/mBio.00229-21 (PMC8092223; doi:10.1128/mBio.00229-21)
Supplement: TABLE S2 [file mBio.00229-21-st002.docx]

**Supplementary Table S2: Strains used in this study.**

| **Strain name** | **Source** | **Parental strain** | **Features** |
| --- | --- | --- | --- |
| WT | Norio Murata (Jap.) | *-* | *Synechocystis* sp. PCC 6803, wild type, glucose-tolerant, non-motile |
| Δ*pirA* | This study | *Synechocystis* sp. PCC 6803 wild type | recombinant *Synechocystis* strain in which the *pirA* gene was deleted and replaced by a kanamycin resistance cassette |
| *pirA*^+^ | This study | *Synechocystis* sp. PCC 6803 wild type | recombinant *Synechocystis* strain carrying the pVZ322-P*petE*:*pirA* plasmid used for ectopic, Cu^2+^-inducible *pirA* expression |
| Δ*pirA* + P*petE*-*pirA* | This study | Δ*pirA* | knockout strain for *pirA* with a P*petE*::*pirA* construct inserted into the chromosome |
